# Polymorphisms in *MMP-1*, *MMP-2*, *MMP-7, MMP-13* and *MT2A* do not contribute to breast, lung and colon cancer risk in polish population

**DOI:** 10.1186/s13053-020-00147-w

**Published:** 2020-07-31

**Authors:** Katarzyna Białkowska, Wojciech Marciniak, Magdalena Muszyńska, Piotr Baszuk, Satish Gupta, Katarzyna Jaworska-Bieniek, Grzegorz Sukiennicki, Katarzyna Durda, Tomasz Gromowski, Marcin Lener, Karolina Prajzendanc, Alicja Łukomska, Cezary Cybulski, Tomasz Huzarski, Jacek Gronwald, Tadeusz Dębniak, Jan Lubiński, Anna Jakubowska

**Affiliations:** 1 Department of Genetics and Pathology, International Hereditary Cancer Center, Pomeranian Medical University, Szczecin, Poland; 2 Read-Gene S.A., Grzepnica, Poland; 3 Strand Life Sciences, Bangalore, Karnataka India; 4 Independent Laboratory of Molecular Biology and Genetic Diagnostics, Pomeranian Medical University, Szczecin, Poland

**Keywords:** Prostate cancer, Matrix metalloproteinases, Metallothioneins, Polymorphisms

## Abstract

**Background:**

Matrix metalloproteinases (MMPs) and metallothioneins (MTs) are Zinc-related proteins which are involved in processes crucial for carcinogenesis such as angiogenesis, proliferation and apoptosis. Several single nucleotide polymorphisms (SNPs) in MMPs and MTs that affect genes expression have been associated with cancer risk, including breast, lung and colon.

**Methods:**

The study group consisted of 648 unselected patients (299 with breast cancer, 199 with lung cancer, 150 with colon cancer) and 648 unaffected individuals. Five SNPs, rs1799750 in *MMP-1,* rs243865 in *MMP-2,* rs11568818 in *MMP-7,* rs2252070 in *MMP-13* and rs28366003 in *MT2A* were genotyped and serum zinc (Zn) level was measured. The cancer risk was calculated using multivariable logistic regression with respect to Zn.

**Results:**

None of the 5 tested polymorphisms showed a correlation with cancer risk in studied groups, although for *MMP-2*, *MMP-7* and *MT2A* non-significant differences in genotypes frequencies among cases and controls were observed.

**Conclusions:**

Analyses of polymorphisms, rs1799750 in *MMP-1*, rs243865 in *MMP-2*, rs11568818 in *MMP-7*, rs2252070 in *MMP-13* and rs28366003 in *MT2A* in relation to serum Zn level did not show significant association with breast, lung and colon cancer risk among polish patients. Further studies are needed to verify this observation.

## Background

Matrix metalloproteinases (MMPs) are a large family of zinc (Zn) containing enzymes which participate in degradation and remodeling of extracellular matrix (ECM) [1]. MMPs are involved in regulation of activity of growth factors, chemokines, cytokines, and other bioactive molecules, thus participate in cell adhesion, proliferation, angiogenesis or apoptosis [2]. Impaired expression of MMPs was reported in various cancers, including breast, colon, esophagus, lung and prostate [3–8]. Upregulation of MMPs in cancer tissue was shown to correlate with breast tumour progression [9], and poor prognosis [10, 11], as well as low survival rate in breast, colon and lung cancer [8, 12, 13].

Metallothioneins (MTs) are group of proteins involved in storage and transport of metals such as Zn, copper and selenium [14, 15]. The property to bind above metals enables MTs to participate in cell growth regulation, proliferation and apoptosis [16]. MTs have also high affinity to bind heavy metals such as cadmium and mercury, thus play a role in protection against toxicity of these metals [14]. The ability to bind reactive oxygen species prevents cells from oxidative DNA damage [17]. Increased MTs levels in cancer cells affect expression of various proteins including p53, caspases, FGF, VEGF, thus may inhibit apoptosis [18, 19] and enhance angiogenesis [20]. Similarly to MMPs, higher expression of MTs in tumors has been shown to correlate with high grade [21] and relapse [22] in breast cancer, as well as progression [23] and low survival [24] in lung cancer patients.

Both MMPs and MTs are Zn-related proteins. MMPs require Zn ions to maintain their structure and functions [1]. MTs are involved in regulation of Zn homeostasis through binding and storing excessive Zn ions when cytosolic Zn level is increased and releasing it in case of Zn deficiency [15, 25]. Zn is one of the most important micronutrient involved in many processes in human body [26] and disruptions in its homeostasis have been linked to several diseases [27–32] including cancers [33–40].

Several single nucleotide polymorphisms (SNPs) in promoter regions of MMPs and MTs genes have been reported to affect gene’s expression [41–46]. Previous reports indicate that SNPs in MMPs and MTs genes may be associated with risk of cancers. Five SNPs, rs1799750 in *MMP-1,* rs243865 in *MMP-2,* rs11568818 in *MMP-7,* rs2252070 in *MMP-13* and rs28366003 in *MT2A* has been studied in various cancers including breast [47–57], lung [47, 58–64] and colon cancer [44, 47, 65–75], but results were ambiguous. The results of meta-analyses suggest that polymorphisms in MMP-1, MMP-2, and MMP-7 may be associated with cancer risk in Asian or Latin-American populations, but not in European or Caucasian populations [63, 76–80]. The MMPs and MTs are strongly associated with Zn homeostasis, so it is highly probable that the influence of SNPs in these genes on cancer risk may be dependent on Zn status. Therefore, it seems highly reasonable to perform multivariable logistic regression analysis to evaluate the association of SNPs in MMPs and MTs together with serum Zn levels with cancer risk. However, such analyses have been not performed to date in any of the previous studies.

The aim of this study was to investigate association of polymorphisms rs1799750 in *MMP-1,* rs243865 in *MMP-2,* rs11568818 in *MMP-7,* rs2252070 in *MMP-13* and rs28366003 in *MT2A*, together with serum Zn level, with occurrence of breast, lung and colon cancer in Poland.

## Material and methods

### Study participants

Study group consisted of 648 unselected patients among whom 299 were diagnosed with breast cancer, 199 with lung cancer and 150 with colon cancer. All patients were diagnosed between 2010 and 2014 at the Clinical Hospital No 2. in Szczecin, had no previous history of malignancy and were not treated prior to blood collection. To each case was matched one cancer free control selected among individuals who took part in a population-based screening study performed in the West-Pomeranian region of Poland to identify familial cancer syndromes. Controls were matched to cases with respect to sex, year of birth (+/− 3 years), occurrence of malignancies among first degree relatives, smoking (status and number of pack/years) and adnexectomy status (for group of breast cancer patients). The characteristics of study groups and controls are shown in Table 1. From all participants enrolled in the study informed written consent for the use of their samples in research was obtained. The study was approved by Ethics Committee of the Pomeranian Medical University in Szczecin, Poland.

**Table 1:** Characteristic of cases and controls included in the study

**Breast cancer group**			
**Characteristic**	**Cases (*n*** **= 299)**	**Controls (*n*** **= 299)**	*p*-value*
Mean year of birth (range)	1953 (1923–1989)	1953 (1923–1990)	0.93
Smoking, n (%)			
Yes (current + past)	129	129	–
No (never smokers)	170	170	–
Pack-years (range)	6.61 (0–40)	6.64 (0–50)	0.49
Adnexectomy status, n (%)			
Yes	15	15	–
No	284	284	–
Occurrence of breast cancer among I° relatives, n (%)			
Yes	39	41	–
No	260	258	–
**Lung cancer group**			
**Characteristic**	**Cases (*n*** **= 199)**	**Controls (*n*** **= 199)**	*p*-value*
Mean year of birth (range)	1947 (1928–1977)	1947 (1931–1976)	0.62
Sex, n (%)			
Females	51	51	–
Males	148	148	–
Smoking, n (%)			
Yes (current + past)	188	183**	–
No (never smokers)	11	16**	–
Pack-years (range)	30.14 (0–150)	29.72 (0–150)	0.50
Occurrence of lung cancer among I° relatives, n (%)			
Yes	37	30	–
No	162	169	–
**Colon cancer group**			
**Characteristic**	**Cases (*n*** **= 150)**	**Controls (*n*** **= 150)**	*p*-value*
Mean year of birth (range)	1947 (1924–1985)	1947 (1927–1984)	0.99
Sex, n (%)			
Females	58	58	–
Males	92	92	–
Smoking, n (%)			
Yes (current + past)	95	94**	–
No (never smokers)	55	56**	–
Pack-years (range)	14.2 (0–90)	14.1 (0–80)	0.30
Occurrence of colon cancer among I° relatives, n (%)			
Yes	17	16	–
No	133	134	–

* *p*-value calculated using Mann-Whitney Test

**controls smoking < 1 year were matched to never smoking cases

### Laboratory analyses

From each person included in the study two peripheral blood samples were collected, one to obtain serum for Zn measurement and second for isolation of genomic DNA. The methods of samples preparation were described previously [81].

Laboratory analyses encompassed measurement of Zn in serum and genotyping of 5 SNPs: rs1799750 in *MMP-1,* rs243865 in *MMP-2,* rs11568818 in *MMP-7,* rs2252070 in *MMP-13* and rs28366003 in *MT2A*, as described previously [81].

### Statistical analysis

Comparison of characteristics between cases and controls was performed using Mann-Whitney U-test. Association of tested SNPs with cancer risk was established by comparing the frequencies of genotypes among patients and healthy individuals in each cancer group. The most frequent homozygous genotype of the analyzed SNP was considered as a ‘reference’. The odds ratios (ORs) and corresponding 95% confidence intervals (95% CI) for each tested SNP were calculated using multivariable logistic regression model and adjusted for serum Zn level. Analyses were performed using R Project for Statistical Computing (ver. 3.3.3.).

## Results

None of the 5 tested polymorphisms showed a correlation with cancer risk in studied groups, although some non-significant differences in genotypes frequencies among cases and controls were observed for *MMP-2*, *MMP-7* and *MT2A*. *MMP-2* TT genotype was less frequent among breast cancer patients comparing to controls (4.3% vs 8.4%) (Table 2), but more frequent in lung cancer cases (5% in cases vs 3% in controls) (Table 3). In colon cancer group we observed that *MMP-7* GG genotype as well as *MT2A* AG and GG genotypes were more frequent among cases than controls (18% vs 13.3 and 12.7% vs 7.3%, respectively) (Table 4).

**Table 2:** Association of analyzed SNPs with breast cancer risk

Genotype	Cases, ***n*** = 299 (%)	Controls, ***n*** = 299 (%)	OR^**a**^ (95%CI)	***p***-value
***MMP-1*(rs1799750)**				
1G/1G	80 (26.8)	80 (26.8)	1	–
1G/2G	140 (46.8)	144 (48.2)	1.01 (0.68–1.46)	0.99
2G/2G	79 (26.4)	75 (25.1)	1.08 (0.70–1.67)	0.73
***MMP-2*(rs243865)**				
CC	172 (57.5)	169 (56.5)	1	–
CT	114 (38.1)	105 (35.1)	1.08 (0.77–1.51)	0.66
TT	13 (4.3)	25 (8.4)	0.56 (0.28–1.11)	0.10
***MMP-7*(rs11568818)**				
AA	106 (35.5)	93 (31.1)	1	–
AG	151 (50.5)	152 (50.8)	0.87 (0.61–1.24)	0.44
GG	42 (14)	54 (18.1)	0.68 (0.42–1.10)	0.11
***MMP-13*(rs2252070)**				
AA	142 (47.5)	146 (48.8)	1	–
AG	133 (44.5)	125 (41.8)	1.12 (0.79–1.59)	0.53
GG	24 (8)	28 (9.4)	0.84 (0.44–1.57)	0.58
***MT2A*(rs28366003)**				
AA	274 (91.6)	267 (89.3)	1	–
AG + GG^b^	25 (8.4)	32 (10.7)	0.73 (0.42–1.27)	0.27

^a^Multivariable logistic regression adjusted for Zn level

^b^Genotypes were summarized because of low number of GG genotype

**Table 3:** Association of analyzed SNPs with lung cancer risk

Genotype	Cases, ***n*** = 199 (%)	Controls, ***n*** = 199(%)	OR^**a**^ (95%CI)	***p***-value
***MMP-1*(rs1799750)**				
1G/1G	48 (24.1)	47 (23.6)	1	–
1G/2G	114 (57.3)	107 (53.8)	0.98 (0.61–1.57)	0.92
2G/2G	37 (18.6)	45 (22.6)	0.77 (0.42–1.39)	0.38
***MMP-2*(rs243865)**				
CC	120 (60.3)	121 (60.8)	1	–
CT	69 (34.7)	72 (36.2)	1.01 (0.65–1.55)	0.98
TT	10 (5)	6 (3)	1.64 (0.56–4.83)	0.37
***MMP-7*(rs11568818)**				
AA	70 (35.2)	71 (35.7)	1	–
AG	98 (49.2)	100 (50.3)	1.03 (0.65–1.61)	0.91
GG	31 (15.6)	28 (14.1)	1.24 (0.63–2.44)	0.54
***MMP-13*(rs2252070)**				
AA	83 (41.7)	96 (48.2)	1	–
AG	99 (49.7)	87 (43.7)	1.38 (0.89–2.14)	0.15
GG	17 (8.5)	16 (8)	1.26 (0.61–2.62)	0.53
***MT2A*(rs28366003)**				
AA	174 (87.4)	180 (90.5)	1	–
AG + GG^b^	25 (12.6)	19 (9.5)	1.52 (0.72–3.18)	0.27

^a^Multivariable logistic regression adjusted for Zn level

^b^Genotypes were summarized because of low number of GG genotype

**Table 4:** Association of analyzed SNPs with colon cancer risk

Genotype	Cases, ***n*** = 150 (%)	Controls, ***n*** = 150 (%)	OR^**a**^ (95%CI)	***p***-value
***MMP-1*(rs1799750)**				
1G/1G	38 (25.3)	49 (32.7)	1	–
1G/2G	75 (50)	66 (44)	1.49 (0.87–2.55)	0.15
2G/2G	37 (24.7)	35 (23.3)	1.34 (0.70–2.56)	0.37
***MMP-2*(rs243865)**				
CC	88 (58.7)	88 (58.7)	1	–
CT	53 (35.3)	54 (36)	1.01 (0.59–1.67)	0.99
TT	9 (6)	8 (5.3)	1.27 (0.46–3.52)	0.65
***MMP-7*(rs11568818)**				
AA	52 (34.7)	53 (35.3)	1	–
AG	71 (47.3)	77 (51.3)	0.96 (0.56–1.65)	0.90
GG	27 (18)	20 (13.3)	1.41 (0.66–2.99)	0.38
***MMP-13*(rs2252070)**				
AA	79 (52.7)	70 (46.7)	1	–
AG	60 (40)	70 (46.7)	0.78 (0.50–1.24)	0.30
GG	11 (7.3)	10 (6.7)	0.95 (0.34–2.64)	0.92
***MT2A*(rs28366003)**				
AA	131 (87.3)	139 (92.7)	1	–
AG + GG^b^	19 (12.7)	11 (7.3)	1.76 (0.81–3.83)	0.15

^a^Multivariable logistic regression adjusted for Zn level

^b^Genotypes were summarized because of low number of GG genotype

## Discussion

In the present study we analyzed whether polymorphisms rs28366003 in *MT2A*, rs1799750 in *MMP-1*, rs243865 in *MMP-2*, rs11568818 in *MMP-7* and rs2252070 in *MMP-13* genes are associated with the risk of breast, lung or colon cancer among polish subjects. Since functions of MTs and MMPs are strongly related to Zn homeostasis, in this study we also measured serum Zn level and performed multivariable analysis adjusted for Zn level. Results did not show any significant correlations with cancer risk in studied groups, although non-significant differences in genotypes frequencies among cancer cases and controls were observed for *MMP-2* in breast and lung cancer group (Tables 2 and 3), *MMP-7* and *MT2A* in colon cancer group (Table 4).

The rs243865 (− 1306 C > T) in *MMP-2* is located in gene promoter and alters the SP-1 transcription factor binding site, resulting in reduction of protein expression [42]. Several studies have examined association of this SNP with the risk of breast, lung and colon cancers, however results were ambiguous. In 2 studies, from Mexico (90 cases and 96 controls) [52] and Saudi Arabia (90 cases and 92 controls) [51], CC genotype was associated with increased risk of breast cancer (OR = 2.15, *p* = 0.01and OR = 2.02, *p* = 0.02 respectively). In analysis of 462 cases and 509 controls from China [50], the T allele was shown to have a protective effect on breast cancer (OR = 0.46, *p* < 0.001). On a contrary, in Greek (113 cases and 124 controls) [53], Brazilian (89 cases and 100 controls) [54] and Swedish (959 cases and 952 controls) [48] studies no association between rs243865 and breast cancer risk was shown. Results of meta-analysis encompassing 9858 cases and 10,871 controls suggest that rs243865 CC genotype is associated with an increased risk of breast cancer only in Latin American population, but not European and Asian [76]. Although in our study we did not found a significant correlation of rs243865 in *MMP-2* with breast cancer risk, the TT genotype found to be less frequent among cases than in controls (4,3% vs. 8.4%, Table 2), what is consistent with the previous findings [50–52]. Analyzes of rs243865 in *MMP-2* in 2 studies conducted in Saudi Arabia comprising 220 cases and 247 controls revealed positive correlation of TT genotype with 6.5- fold increased risk of colon cancer [67, 71]. However, results of 3 other studies performed in Chinese, Swedish and Korean populations did not confirm this observation [68–70]. Association of rs243865 with 2-fold increased lung cancer risk was observed in 2 Chinese analyses encompassing of 781 cases, 852 controls (OR 2.18; 95% CI 1.70–2.79) and 770 cases, 777 controls (OR 2.12; 95% CI 1.64–2.72) [59, 60]. Although, in 2 other studies, from France (90 cases and 90 controls) [61] and Turkey (200 cases and 100 controls) [62], no correlation with lung cancer risk was observed. Results of 2 meta-analyzes indicate that rs243865 in *MMP-2* correlates with lung cancer risk in Asian populations, but not Caucasians [77, 78]. In our study we did not detect significant association of rs243865 in *MMP-2* with lung and colon cancer risk, however we observed higher frequency of TT genotype in lung cancer patients comparing to controls (5% vs. 3%, Table 3).

The SNP rs11568818 (− 181 A > G) in *MMP-7* gene has been shown to affect gene expression and G allele was found to upregulate the transcription [43, 82]. The rs11568818 has been analyzed previously in relation to colon, lung and breast cancers, but results were inconclusive. In a Polish study encompassing 184 cases and 205 controls, the GG genotype was more frequent among cancer patients than healthy individuals (28% vs. 22%) and correlated with 2-times increased colon cancer risk (OR = 2.12, *p* = 0.018) [73]. Accordant results were presented in Italian study of 58 cases and 118 controls showing similarly higher frequency of GG in cancer patients comparing to controls (26% vs. 13%) and association of GG with almost 2.5-time higher colon cancer risk (OR = 2.41; *p* = 0.03) [72]. On a contrary, in a Brazilian study of 130 colon cancer cases and 130 controls, no association of rs11568818 in *MMP-7* with colon cancer risk was shown [74]. In our analysis of 150 colon cancer cases and 150 controls we did not detect a significant association of the rs11568818 with cancer risk, however we observed higher frequency of GG genotype in cancer patients than in healthy individuals (18% vs. 13.3%, Table 4), what is consistent with previous Polish [73] and Italian [72] observations. Analyses of rs11568818 in *MMP-7* performed in Chinese population suggested that rs11568818 may correlate with 2-fold increased risk of lung cancer (243 cases and 350 controls, OR = 2.0, 95% CI = 1.23–3.24 for AG and GG genotypes) [83], but not breast cancer (1079 cases and 082 controls) [55]. A meta-analysis of 24 studies encompassing 12 populations and including 10 different cancer sites revealed that *MMP-7* SNP may be associated with the risk of various cancers, including gastric, colon, liver, bladder, cervix, ovary, breast, brain and lung, but only in Asian population (OR = 2.18, 95%CI 1.68–2.84) and not European (OR = 1.07, 95% CI 0.6–1.89) [79]. In our analysis the rs11568818 was not associated with the risk of breast and lung cancers.

The promoter polymorphism rs28366003 (− 5 A > G) in *MT2A* gene is suggested to reduce the heavy metal induced *MT2A* transcription, what could result in increased toxicity of heavy metals [45, 46]. Several analyzes suggested that, the rs28366003 in *MT2A* correlates with breast cancer risk. In a Polish study including 534 cases and 556 controls rs28366003 was shown to be associated with almost 2-fold increased cancer risk for AG-heterozygotes and GG-homozygotes (OR = 1.93; *p* = 0.02) [56]. Similarly in a Chinese study conducted in a group of 459 breast cancer patients and 549 controls, rs28366003 was found to correlate with over 2-fold higher breast cancer risk for AG and GG genotypes (OR = 2.66, *p* < 0.0001) [57]. In our study we did not detect significant correlation of rs28366003 in *MT2A* with breast, colon or lung cancer risk, although we observed higher frequency of AG and GG genotypes in colon and lung cancer cases in comparison to controls (12.7% vs. 7.3 and 12.6% vs. 9.5%, respectively).

The rs1799750 in *MMP-1* (− 1607 1G > 2G) is an insertion polymorphism located in promoter region and 2G allele was found to upregulate gene expression [84]. Results of 3 studies performed in Sweden (959 cases and 952 controls) [48], Italy (43 cases and 164 controls) [47] and Poland (270 cases and 300 controls) [49] showed no association of this polymorphism with breast cancer risk. Analysis performed in USA encompasing1752 cases and 1363 controls revealed association of rs179975 with lung cancer risk but only in men and never-smokers [58]. However, Italian study of 29 cases and 164 controls did not confirm this observation [47]. Results of two separate meta-analyzes, each including 9 studies, indicated that rs1799750 in *MMP-1* may be associated with lung cancer risk, but only in Asian populations [63, 80]. In two other studies, from Japan (101 cases and 127 controls) and Italy (63 cases and 164 controls), rs179975 was suggested to correlate with colon cancer risk. It was found that comparing to 1G/1G genotype, 2G/2G genotype was associated with 2-fold increased colon cancer risk (OR = 2.08; *p* = 0.007 and OR = 2.21; *p* = 0.014, respectively) [65, 66]. However, other Italian study comprising 60 cases and 164 controls showed no association of this SNP with colon cancer [47]. In our analysis we did not observe significant association of rs1799750 in *MMP-1* with breast, lung and colon cancer risk.

The SNP rs2252070 (− 77 A > G) in *MMP-13* is located in promoter region and is suggested to affect gene expression [85]. Few studies have investigated the correlation rs2252070 with cancers. No association of rs2252070 was detected with lung cancer risk in Spanish study of 501 cases and 506 controls [64], as well as with colon cancer risk in Swedish analysis of 385 cases and 619 controls [44]. On a contrary, a Mexican study encompassing 102 cases and 121 controls showed association of AA and AG genotypes with increased colon cancer risk (OR = 3.4, p = 0.01) [75]. In our analysis we did not observe correlation of rs2252070 in *MMP-13* with breast, lung or colon cancer risk.

In our study of 1296 individuals including 648 cancer patients (299 diagnosed with breast cancer, 199 with lung cancer, 150 with colon cancer) and equal number of healthy controls we were unable to detect a significant correlation of polymorphisms rs1799750 in *MMP-1,* rs243865 in *MMP-2,* rs11568818 in *MMP-7,* rs2252070 in *MMP-13* and rs28366003 in *MT2A* with occurrence of breast, lung and colon cancer in Poland. As functions of matrix metalloproteinases (*MMP-1*, *MMP-2*, *MMP-7*,*MMP-13*) and metallothioneins (*MT2A*) are strongly related to Zn homeostasis, we measured serum Zn level and performed multivariable analysis of tested SNPs adjusted for Zn level, which is the main advantage of our study. Such approach allowed reducing the risk of bias caused by Zn level, which might be significant factor influencing functions of MTs and MMPs and affecting cancer risk. Zn level was not considered in previous studies investigating association of *MMP-1, MMP-2, MMP-7, MMP-13* and *MT2A* polymorphism with cancer risk. In addition to tested serum Zn level we also used strong pairing criteria for cases and controls. To each affected individual one control was matched with respect to significant factors such birth year, family history (number of cancers in first degree relatives), smoking and adnexectomy status (in breast cancer group) to avoid influence of these factors on cancer risk.

In our study, despite the fact that sample size was larger than in several previously published analyzes [47, 51–54, 61, 62, 65–71, 75], we were unable to detect significant result, what could be an effect of insufficient study sample. Further studies are needed to verify association of rs1799750 in *MMP-1,* rs243865 in *MMP-2,* rs11568818 in *MMP-7,* rs2252070 in *MMP-13* and rs28366003 in *MT2A* with breast, colon and lung cancers.

## Conclusion

Analyses of polymorphisms rs1799750 in *MMP-1,* rs243865 in *MMP-2,* rs11568818 in *MMP-7,* rs2252070 in *MMP-13* and rs28366003 in *MT2A* in relation to serum Zn level did not show significant association of tested SNPs with breast, lung and colon cancer risk among polish patients.

## Data Availability

All relevant data are within the paper and its supporting information files.

## Abbreviations

SNP: Single nucleotide polymorphism
PCR: Polymerase chain reaction
MMP: Matrix metalloproteinase
MT: Metallothionein
